# Development and implementation of a nurse-led allergy clinic model in primary care: feasibility trial protocol

**DOI:** 10.1038/s41533-019-0155-5

**Published:** 2019-12-06

**Authors:** Margaret Kelman, Victoria Hammersley, Marilyn Kendall, Mome Mukherjee, Lynn Morrice, Susan Harley, Jürgen Schwarze, Aziz Sheikh

**Affiliations:** 1 Usher Institute, The University of Edinburgh, Old Medical School Teviot Place, Edinburgh, EH8, 9AG UK; 2 Centre for Inflammation Research, The University of Edinburgh, The Queen’s Medical Research Institute, Edinburgh, EH16, 4TJ UK

**Keywords:** Health care, Diseases

## Abstract

In the United Kingdom, there are acknowledged short comings in allergy care provision for patients seen in primary care. There is a lack of allergy training for healthcare professionals and this leads to inappropriate referrals to the limited number of allergy specialists. The primary aims of this study are to assess the feasibility of delivering and evaluating a new nurse-led allergy service in primary care, measured by recruitment, retention and quality of life. This is a single arm feasibility trial in which up to 250 participants referred to the nurse-led allergy clinic will receive the intervention and complete 6–12 weeks follow-up before being referred back to their usual care. Primary outcomes for this study will be establishment of clinics, recruitment and retention rates, and estimates of change in disease-specific quality of life measures. Secondary outcomes will be acceptability of the new service to participants/carers and healthcare professionals. A sample of participants and professional stakeholders will take part in more in-depth semi-structured qualitative interviews. Data from this feasibility trial will be used to inform plans for a pilot randomised controlled trial of nurse-led allergy clinics.

## Introduction

The incidence and prevalence of allergic diseases has increased substantially over recent decades, and the United Kingdom has one of the highest rates of allergy in the western world.^1^ Allergic conditions affect approximately one in three people in Scotland at some point in their lives^2^ and the prevalence is thought to be even higher in children and young people, with an estimated 50% of the population now affected.^3^ Allergic conditions cost the UK’s national health services over £1 billion per annum.^4^

The term allergy covers a broad range of clinical conditions that share a common pathophysiology. The most common conditions and those responsible for the greatest healthcare utilisation include atopic eczema/dermatitis (henceforth atopic eczema), food allergy, asthma, allergic rhinitis, allergic conjunctivitis, urticaria and anaphylaxis. The majority of cases of allergy in the United Kingdom are managed within primary care.^5^ Specialist allergy care provision is patchy, but where it exists, evidence suggests that there are often unnecessary referrals to secondary care for conditions that could be dealt with in primary care settings.^5^ This in turn reflects important gaps in undergraduate and postgraduate training of general practitioners (GPs).^6^ There have been a number of national calls and initiatives aimed at improving allergy care provision in the United Kingdom.^7–9^

Jutel et al.^10^ have demonstrated that, given the very large numbers of people now affected by allergic conditions, expansion of allergy services delivered within ‘specialist settings’ is not a viable proposition. Rather, they suggest that there is a need to develop provision of allergy care within community settings. A report by the Children’s and Young People’s Allergy Network Scotland in 2013 found that, across Scotland, primary care practitioners did not feel they have the skills or knowledge to provide good quality allergy care, especially around diagnostic testing for allergy, and the ability to interpret the results.^11^

An earlier pilot primary care-based allergy service in England, run by a specialist allergy nurse and a GP with a special interest in respiratory disease and allergy, showed that a primary care intervention for allergy could effectively deal with the majority of cases of allergy seen in primary care, resulting in a reduction in inappropriate referrals into secondary care, an increase in self-supported care for patients and a saving in costs.^12^ These data however come from an uncontrolled study and therefore need to be interpreted with caution. A more recent study by Smith et al.^13^ found that a large percentage of referrals, which would have otherwise have been seen in secondary care, could adequately be dealt with in primary care by a practitioner with a specialist interest in allergy. Findings from a recent systematic review support the need for alternative models of allergy care provision.^14^ Systematic reviews of disease-specific nurse-led clinics have shown high levels of patient satisfaction; however, there remains a need for experimental trials to improve the level of evidence.^15,16^ In response to these findings, we are proposing a novel allergy service intervention that is nurse-led and primary care-based to improve the patient pathway and access to early allergy diagnosis and management.

The primary aims of this trial are to assess the feasibility of delivering and evaluating a nurse-led allergy clinic in a primary care setting in Scotland and to estimate the impact on disease-specific quality of life in patients referred to the service. The secondary aim is to measure the acceptability of the service to patient/carers and healthcare professionals (HCPs).

## Discussion

At the time of submission, we have recruited two hub practices who are receiving referrals from 21 of the 37 spoke practices, and one practice which is hosting a clinic for their own patients. There have been 265 referrals to the service, and 214 have been seen in the nurse-led clinic. Recruitment started in July 2017 and will continue until January 2020, when it is anticipated we will have reached our target sample size of 250 patients who have completed study follow-up.

## Methods

### Design

This is a feasibility trial of a single group assigned to a nurse-led allergy clinic in primary care. The trial was registered on ClinicalTrials.gov reference NCT03826953 on 1 February 2019 (https://clinicaltrials.gov/ct2/show/NCT03826953?term=NCT03826953&rank=1).

### Setting

Nurse-led allergy clinics are being established in the South West and South East localities of the Edinburgh Health and Social Care Partnership, Scotland.

### Ethical approval

South East Scotland Research Ethics committee approval was obtained (REC ref: 17/SS/0057).

### Recruitment of practices

A hub and spoke model was chosen for this trial, where hub practices act as the host for the nurse-led allergy clinic and spokes are the practices that can refer patients to these hubs. Eight general practices in the Edinburgh Health and Social Care Partnership newly formed south-east area cluster were approached by email to be hubs. This newly established cluster group has 20 general practices serving an estimated population of 134,000 patients, with a range of patient demographics. Two practices expressed an interest and were visited by the allergy nurse to explain the requirements for hosting the clinics. One practice agreed to operate as a hub practice, accepting referrals from spoke practices in the cluster, and the other agreed to host the clinics, but accepting only internal referrals. Subsequently, a third hub practice was recruited in the south-west area of Edinburgh with 17 practices acting as spokes serving an estimated population of 135,000 patients. Locality managers distributed study information packs to all practices in their cluster, and this was then followed up with an email to individual clinicians detailing the inclusion criteria for referrals, and a referral proforma. Regular emails are sent out reminding clinicians of the nurse-led allergy service.

### Recruitment of participants

All adults, young people and children who fit the eligibility criteria from the spoke practices can be referred to the nurse-led allergy clinic. Referrals are accepted from all HCPs who manage patients with allergies, including GPs, practice nurses and health visitors. The HCP will select the appropriate patients for referral, using an inclusion criteria checklist, and ask if they wish to attend the allergy clinic, advising patients and their carers that the nurse-led allergy clinic is part of a research study. On attendance at the clinic, patients/patient’s guardian are advised that they will need to provide written consent to take part in the study. If the patient does not wish to take part in the research project, they will be seen by their HCP as usual.

### Eligibility criteria

The inclusion criteria are:

- Children aged <36 months with suspected food allergy.
- Children aged <36 months with moderate-to-severe atopic eczema not responding to standard treatment.
- Children and young people up to 16 years of age with suspected allergic rhinitis symptoms are not responsive to a combination of oral antihistamines and nasal steroids.
- Young people and adults (from 16 years of age) with a history of anaphylaxis or suspected anaphylaxis.
- Able to give informed consent.

The exclusion criteria are:

- Children aged <36 months with suspected or confirmed non-IgE-mediated food allergy presenting primarily with gastrointestinal symptoms.
- Single urticarial reactions without obvious triggers.
- Non-allergic chronic urticaria.
- Drug allergy.
- Well-controlled allergic rhinitis, asthma or atopic eczema.
- Mild-to-moderate atopic eczema without any obvious allergic trigger.
- Localised insect sting reactions.
- Unable to give informed consent.

### The intervention

**Fig. 1:**
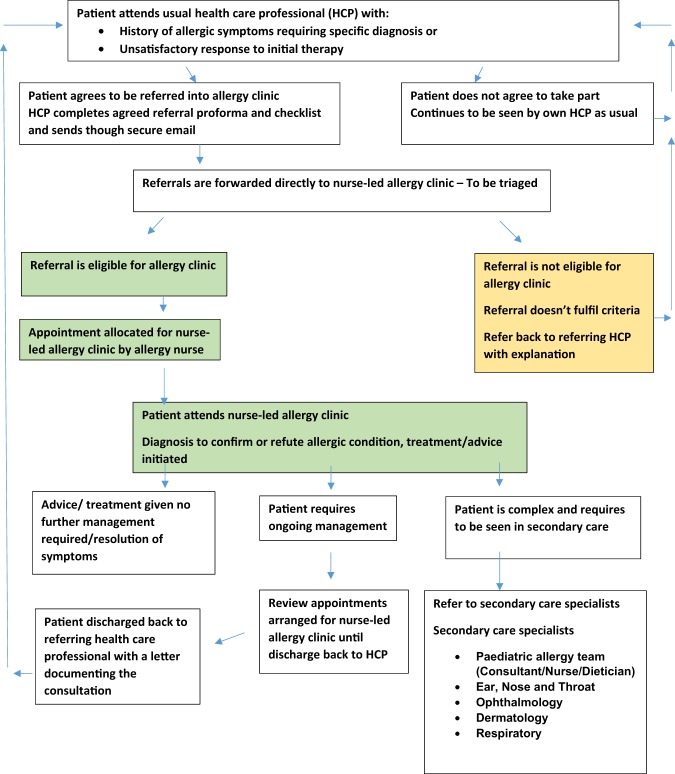
Referral process into the nurse-led allergy clinic.

The intervention is a nurse-led allergy service in primary care. The nurses have a postgraduate qualification in allergy and extensive experience of secondary care allergy clinics and are supported when necessary by a team of specialist services, including paediatric and adult allergy, dermatology, ear, nose and throat, respiratory, and ophthalmology. Referring HCPs are asked to complete a referral proforma and a referral sheet for each eligible patient, which is sent via secure National Health Service (NHS) email to the allergy nurse. Referrals are triaged by the allergy nurse according to the inclusion/exclusion criteria, and allocated an appointment by phone and letter/email. The ~45-min appointment includes an explanation of the study, taking written consent, and completion of baseline questionnaires assessing disease-specific quality of life and costs incurred by the patients/carers (see outcome measures below). The nurse then completes an allergy-focused history and clinical examination. Following this, the nurse will carry out investigations such as skin prick testing, give advice (written and oral) to support the management of allergies, including demonstration of medical devices (e.g. adrenaline auto-injectors, inhalers) and, if required, recommend medication(s). A letter summarising the consultation is then sent to the referring HCP including details of the medication to prescribe and the rationale for prescribing these items. The participants are then discharged back to the care of their referring HCP once they have seen the allergy nurse and have a diagnosis confirmed or refuted (see Fig. 1).

**Fig. 2:**
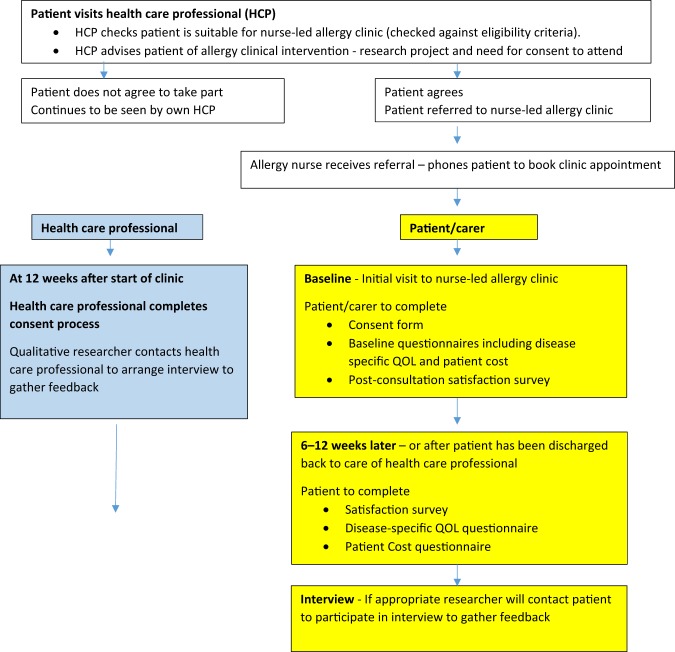
Data collection.

If, on examination, the allergy nurse feels the participant has multiple severe allergies or requires specialist hospital-based assessment, the participant will be advised that they will require referral onto secondary care (Fig. 2). Where appropriate, the allergy nurse will refer participants directly to specialists within secondary care. Where the local policy will not support nurse-led referrals, the nurse practitioner will make recommendations to the GP for the referral of the participant to the appropriate specialist services. Participants who have been seen in secondary care will be referred back to their usual HCP for long-term management. Long-term clinical follow-up will not be offered; some short-term follow-up may, however, be offered if deemed appropriate. All participants will return to their usual HCP for follow-up and long-term management.

### Adverse events

The allergy nurses will ensure that when performing skin prick testing, rescue medications are available at the time of the clinic in the unlikely event of a medical emergency. In the case of an adverse event, the allergy nurse has a responsibility to attend to the safety of the participant and report the incident as soon as is practicable to the on-call GP and practice manager and follow local reporting procedures within the practice. The allergy nurse will also keep a log of any adverse event or adverse reactions. Any serious adverse event or reactions will be reported immediately to the Academic and Clinical Central Office for Research and Development^17^ following the standard operating procedure.

### Outcome measures

This feasibility trial focuses on the ability to set up a new service in primary care, with a novel ‘hub and spoke’ referral model for patients with allergic disease. Prior to a pilot randomised controlled trial, it is important to assess the ability to recruit patients via this new service, and establish recruitment and retention rates and questionnaire response rates for future sample size estimates. The primary outcomes are:

- Recruitment of practices to facilitate establishing the new service and make referrals.
- Referral and consultation rates for nurse-led allergy clinic and retention rates.
- The change in disease-specific quality of life questionnaires between baseline and 6–12 weeks post intervention.

The following disease-specific quality of life questionnaires will be completed at baseline prior to the clinical intervention and at 6–12 weeks post intervention by telephone or email, and face to face for those patients who require a follow-up clinic appointment.

- Dermatology
  - ○ Infants’ Dermatitis Quality Of Life Index^18^
- Allergic rhinitis
  - ○ Mini Rhinitis Quality of Life Questionnaire^19^
- Food allergy and anaphylaxis
  - ○ Food Allergy Quality of Life Questionnaire – Parent Form (children aged 0–12)^20^
  - ○ Food Allergy Quality of Life Questionnaire – Teenager Form (13–17 years)^21^
  - ○ Food Allergy Quality of Life Questionnaire – Adult form^22^

The secondary outcomes are:

- Patient satisfaction, measured immediately post intervention and 6–12 weeks post intervention by questionnaire.
- Referring HCP satisfaction measured after 1 year of the start of the nurse-led allergy clinics by questionnaire.
- Costs incurred by patients/carers.

A subset of referring HCPs, practice managers and patients/carers will be invited to take part in a semi-structured interview to gain in-depth insight into the feasibility of setting up and referring to the clinic, and to explore expectations and experiences of attending the nurse-led primary care allergy clinic.

### Sample size

The sample size for this feasibility trial was informed by an English pilot study,^12^ which over a 9-month period recruited 141 patients to a primary care allergy clinic. We estimate that, taking account of the available time and resources for this study, ~250 patients with completed study follow-up will provide data to inform deliberations to proceed to a pilot randomised controlled trial.

We will interview up to ten HCPs/professional stakeholders, and 20 patients/carers who attend the nurse-led allergy clinic, to cover the experiences of patients and carers with a range of ages and allergic conditions.

### Descriptive analysis

We will describe the number of practices approached to act as hubs and spokes and the number recruited. The characteristics of referring practices in terms of number of patients referred, practice list size and deprivation score will be reported. For patients we will describe the number approached and recruited, age, sex and referring condition. We will report the retention rate for follow-up. For disease-specific quality of life, the difference in the disease-specific quality of life score at baseline and post intervention will be descriptively compared without any formal statistical testing. Patient and professional satisfaction with the service will be reported. Costs (such as travel and time off work) incurred by the patients/carers will be reported.

### Qualitative analysis

Interviews will be digitally recorded, transcribed verbatim and anonymised and transferred to NVivo (version 11) for coding. The thematic analysis^23^ will be iterative and ongoing, in that insights gained from the early interviews will be used to guide data generation in later interviews. The formal thematic coding will be framed around both the research questions and themes arising from the data. Ma.K. will code all the transcripts and the evolving analysis will be reviewed by the study group at their quarterly meetings in order to include a range of interpretative perspectives.

## Supplementary information

Patient satisfaction questionnaire
