# Supplementary material for: Development and implementation of a nurse-led allergy clinic model in primary care: feasibility trial protocol
Source: NPJ Prim Care Respir Med. 2019 Dec 6;29:44. doi: 10.1038/s41533-019-0155-5 (PMC6897953; doi:10.1038/s41533-019-0155-5)
Supplement: Supplementary file 1 — Patient satisfaction questionnaire [file 41533_2019_155_MOESM1_ESM.docx]

**Patient satisfaction questionnaire**

**Nurse Led Allergy Clinic**

**Participant Satisfaction Questionnaire**

We are currently trying to improve the services for patients with allergic conditions, and require your help. We have set up a new GP allergy clinic, which will run in your community for approx. 2 years. As you have attended this clinic, please complete the following questionnaire and post it in the box available. You are under no obligation to complete a questionnaire and your decision not to do so will not affect your future management in any way.

Thank you for your co-operation.

Please mark (x) the appropriate box.

**1.** Since attending the allergy nurse clinic, is your allergic condition?

| Worse: | Slightly worse: | No change: | Slightly better: | Much better: |
| --- | --- | --- | --- | --- |

**2.** How easily were you able to keep to your personal management /treatment plan advised by the allergy nurse?

| Very easily: | Fairly easily: | No problem: | Some difficulty: | Great difficulty: |
| --- | --- | --- | --- | --- |

If difficulties were encountered please explain:

|  |
| --- |

**3.** During your recent visits to the surgery your treatments and care were managed by an Allergy Nurse Practitioner. We would like to know of your experience of this new service. Please answer the following questions:

**3a.** Did you receive prior notification that you would be attending a nurse led clinic? **Yes No**

**3b.** On a scale of 1-5 how happy were you with the following aspects of your allergy care? (1= not happy; 5 = very happy)

Length of time from last seeing your GP to being seen in the clinic: 1 2 3 4 5

Examination 1 2 3 4 5

Discussion with you of treatment options 1 2 3 4 5

Discussion with you of how to use the treatments 1 2 3 4 5

General information given by the nurse 1 2 3 4 5

Ease of getting your prescription 1 2 3 4 5

How well the treatment worked 1 2 3 4 5

Any other comments:

|  |
| --- |

**4.** What was the best part of your treatment and/or attendance at the nurse led clinic?

|  |
| --- |

**5.** What was the worst part of your treatment and/or attendance at the nurse led clinic?

|  |
| --- |

**6.** Next time you visit for a review of your allergic condition, how happy would you be to be seen in the nurse led clinic? (1=not happy; 5 = very happy)

1 2 3 4 5

**7.** The nurse led clinic also operates a telephone follow-up service for patients. Were you aware of the service? **Yes No**

If **Yes,** is this a service you have used

If **Yes,** on a scale of 1 -5, how happy were you with the service given?

1 2 3 4 5

**8.** We are always trying to improve the services in your community for patients. The allergy clinic is a 2 year pilot project and will be monitored to determine the future of the service. Your comments will be helpful. Please feel free to make any suggestion:

|  |
| --- |

**9.** Please make further comments regarding your treatment, care, or the nurse led clinic in the space below:

|  |
| --- |

**Thank you for taking the time to complete this questionnaire. Your views are important to us and will be taken into consideration when making any changes to services.**

**HCP satisfaction questionnaire**

**Nurse Led Allergy Clinic**

**HCP Satisfaction Survey**

The new nurse led allergy clinic has been serving your patients and community for the last six weeks. As part of an audit we would be interested in your opinion as to how this has affected services and the management of care for your patients.

Thank you for your co-operation.

Please mark (x) the appropriate box.

**1.** Please estimate how many patients with an allergy have you referred to the allergy nurse practitioner in the last six weeks?

| 0 | 1 -10 | 11 - 20 | 21- 30 | More than 30 |
| --- | --- | --- | --- | --- |

**2.** How easily were you able to refer/access the service for these patients?

| Very easily: | Fairly easily: | No problem: | Some difficulty: | Great difficulty: |
| --- | --- | --- | --- | --- |

If difficulties were encountered please explain:

|  |
| --- |

**3.** Your patients’ treatments and care were managed by an allergy nurse practitioner. We would like to know of your experience of this new service. Please answer the following questions:

**3a.** Did you receive prior notification of dates, times, venues of the clinics which are run by the allergy nurse practitioner:

**Yes/ No (please circle)**

**3b.** Generally, how happy were you with the following aspects of your patients’ care? (1= not happy; 5 = very happy)

Length of time from seeing you to being seen in the clinic: : 1 2 3 4 5

Examination / assessment of your patients condition: 1 2 3 4 5

Appropriateness of treatments recommended by nurse: 1 2 3 4 5

Documentation of how to use the treatments: 1 2 3 4 5

Ease for patient to get prescription: 1 2 3 4 5

Cost effectiveness of the treatment regimens: 1 2 3 4 5

Clinical effectiveness of the treatment regimens: 1 2 3 4 5

Any other comments:

|  |
| --- |

**4.** What is the best aspect of the allergy nurse led clinic?

|  |
| --- |

**5.** What was the worst aspect of the allergy nurse led clinic?

|  |
| --- |

**6.** Next time you consult a patient with an allergic condition, how happy would you be to be for them to be seen in the nurse led clinic?

(1 = not happy 5= very happy) 1 2 3 4 5

**7.** The nurse led clinic also operates a telephone follow-up for patients. Were you aware of the telephone follow up service?

**Yes No**

If **Yes,** how useful has the follow-up telephone service been for you and your patients?

(1 = not useful; 5 very useful) 1 2 3 4 5

1. If the allergy nurse led service **had not** been available:

Please estimate what percentage of the patients, seen by the nurse practitioner, would you have referred onto secondary care?

| None | 1 - 25% | 26 - 50% | 51 - 75% | 76 – 99% | 100% |
| --- | --- | --- | --- | --- | --- |

**9.** We are trying to improve the allergy services provided for patients. Please make any suggestions:

|  |
| --- |

**10.** Please make further comments regarding the treatment and care at the nurse led clinic in the space below:

|  |
| --- |

**Thank you for taking the time to complete this questionnaire. Your views are important to us and will be taken into consideration when making any changes to services.**
